# Comprehensive analysis of alternative splicing in rice and comparative analyses with Arabidopsis

**DOI:** 10.1186/1471-2164-7-327

**Published:** 2006-12-28

**Authors:** Matthew A Campbell, Brian J Haas, John P Hamilton, Stephen M Mount, C Robin Buell

**Affiliations:** 1 The Institute for Genomic Research, 9712 Medical Center Drive, Rockville, Maryland 20850, USA; 2 Center for Computational Bioinformatics and Computational Biology and Department of Cell Biology and Molecular Genetics, University of Maryland, College Park, Maryland, 20742, USA

## Abstract

**Background:**

Recently, genomic sequencing efforts were finished for *Oryza sativa *(cultivated rice) and *Arabidopsis thaliana *(Arabidopsis). Additionally, these two plant species have extensive cDNA and expressed sequence tag (EST) libraries. We employed the Program to Assemble Spliced Alignments (PASA) to identify and analyze alternatively spliced isoforms in both species.

**Results:**

A comprehensive analysis of alternative splicing was performed in rice that started with >1.1 million publicly available spliced ESTs and over 30,000 full length cDNAs in conjunction with the newly enhanced PASA software. A parallel analysis was performed with Arabidopsis to compare and ascertain potential differences between monocots and dicots. Alternative splicing is a widespread phenomenon (observed in greater than 30% of the loci with transcript support) and we have described nine alternative splicing variations. While alternative splicing has the potential to create many RNA isoforms from a single locus, the majority of loci generate only two or three isoforms and transcript support indicates that these isoforms are generally not rare events. For the alternate donor (AD) and acceptor (AA) classes, the distance between the splice sites for the majority of events was found to be less than 50 basepairs (bp). In both species, the most frequent distance between AA is 3 bp, consistent with reports in mammalian systems. Conversely, the most frequent distance between AD is 4 bp in both plant species, as previously observed in mouse. Most alternative splicing variations are localized to the protein coding sequence and are predicted to significantly alter the coding sequence.

**Conclusion:**

Alternative splicing is widespread in both rice and Arabidopsis and these species share many common features. Interestingly, alternative splicing may play a role beyond creating novel combinations of transcripts that expand the proteome. Many isoforms will presumably have negative consequences for protein structure and function, suggesting that their biological role involves post-transcriptional regulation of gene expression.

## Background

Cultivated rice (*Oryza sativa*) is considered a model for species within the Poaceae family and, in particular, for agronomically important cereals such as maize (*Zea mays*), wheat (*Triticum aestivum*), and barley (*Hordeum vulgare*). Recently, map-based sequencing of a *japonica *subspecies of rice has been completed with the final sequence representing ~95% of the estimated 389 Mb genome [1]. In addition to having a nearly complete genomic sequence, rice has 33,799 full-length cDNA (FL-cDNAs) sequences as well as a collection of >1.15 million publicly available expressed sequence tags (ESTs) and cDNAs [2,3]. The rice genome assembly, based upon the TIGR annotation effort [4], contains 12 pseudomolecules totaling ~372 Mb of sequence and 55,890 genes (of which 13,327 are identified as transposable element (TE)-related).

In this study, we performed a comprehensive analysis of alternative splicing in rice, a model monocotyledonous species, using the latest set of rice transcript data and the Program to Assemble Spliced Alignments (PASA). The PASA software [5] assembles and clusters spliced transcript alignments, providing transcript-based gene structures that are used to automatically improve existing gene annotations by adding untranslated regions (UTRs), adjusting intron and exon boundaries, and adding new models that represent alternative splicing, among its numerous other functions. Alignment assembly, as performed via PASA, is particularly well-suited to the study of alternative splicing. PASA assembles overlapping and compatible alignments into maximal alignment assemblies; compatible alignments are defined as overlapping alignments that are transcribed on the same strand and have identical introns in their regions of overlap. If all overlapping transcripts yield consistent gene structures, they are assembled into a single alignment assembly, and by doing so, PASA acts to simply consolidate the transcript alignments into a single gene structure. However, overlapping transcript alignments that have different introns in their region of overlap, due to a splicing variation, are found incompatible and cannot be assembled together, and hence occupy distinct maximal alignment assemblies. After assembling all overlapping alignments, those distinct PASA assemblies found overlapping at the same locus and transcribed in the same orientation yield representative splicing isoforms for that locus.

Many algorithms have been introduced to reconstruct gene structures from spliced alignments of transcripts to genome sequences [6-9]. These most often decompose the alignments into introns and exons, build a splicing graph data structure [10] and traverse the graph to find combinations of introns and exons that are best supported by the underlying data under some scoring scheme. An inherent danger of splicing graphs is that they have the potential to report combinations of introns and exons for which there is no evidence in existing transcript alignments. Alternative strategies to reconstruct isoforms assemble individual whole alignments instead of their component introns and exons, and by doing so, retain distal correlations between non-adjacent exons found within single transcript alignments [5,11,12]. Isoform reconstruction (alignment assembly) as performed by PASA is strictly an exercise in dynamic programming to chain (assemble) together a compatible series of whole transcript alignments such that each alignment is found within a maximal chain of compatible alignments [5]. This generates an isoform structure supported by the largest number of compatible whole EST and whole cDNA alignments.

Our comprehensive analysis confirms that alternative splicing is a widespread phenomenon, and also confirms prior research into alternative splicing in both rice and Arabidopsis [5,13-17] The most recent analysis for these species identified 14,452 alternative splicing events affecting 6,586 rice genes and 8,264 alternative splicing events affecting 4,707 Arabidopsis genes [18]. Our analysis incorporates new data to extend these numbers to 36,650 events affecting 8,772 rice genes and 16,252 events affecting 5,313 Arabidopsis genes. We also note some parallels with alternative splicing in mammalian species. For example, alternate acceptor (AA) splice sites for these two plant species are most often separated by only three base pairs (bp); a feature observed in mammalian species [19-22]. However, when looking at the AA class overall for rice and Arabidopsis, the majority of these events are predicted to result in a downstream frameshift in translation, consistent with research in mouse [22]. Alternate donor (AD) splice sites are most often found to be separated by only four bp in both species, and this maximal occurrence was previously observed in mouse with the majority of the AD isoforms yielding a frameshift [23]. The retained intron (RI) class of alternative splicing is prevalent in both rice and Arabidopsis and often introduces a premature stop codon in the intron. These three classes (AA, AD, and RI) represent the majority of the alternative splicing events in both rice and Arabidopsis with the occurrence of the remaining classes being far less frequent. Further, the frequent introduction of either a frameshift or premature stop codon by the three predominant classes of splicing events suggests that post-transcriptional regulation via nonsense mediated decay (NMD) may play a more important role than augmenting the proteome.

## Results

### PASA-generated transcript alignment assemblies for rice and Arabidopsis

All ESTs and mRNA (mRNAs include full-length cDNAs (FL-cDNAs)) sequences were obtained from public databases [2,3]. These data, current as of February 15, 2006 include 1,156,705 rice ESTs, 35,078 rice mRNAs, 690,119 Arabidopsis ESTs, and 66,642 Arabidopsis mRNA sequences. The number of rice ESTs is nearly double that of Arabidopsis due to 778,739 rice ESTs that were deposited in DDBJ by the RIKEN-based Full-length cDNA Consortium. Additionally, the RIKEN effort has generated >28,000 rice FL-cDNAs that are publicly available [14]. While Arabidopsis has fewer total ESTs (Table 1), it has nearly double the number of FL-cDNAs. Approximately 96–98% of all transcripts were found to match their respective genomes using the GMAP transcript alignment software [24].

We limited our analysis of alternative splicing to only those transcript alignments that have a nearly perfect alignment to the genome (e.g. 90% of their length at greater than 95% identity). Our overall averages for length and identity exceeded 99% (data not shown). Additionally, all exon boundaries required canonical splice sites (e.g. GT-AG, GC-AG, AT-AC) [25-29]. Finally, since we are focusing on alternatively spliced transcripts, it is essential that we have high confidence in the mapping of the exon-intron boundaries. We have found that spliced alignment programs will sometimes introduce gaps and mismatches within the exon sequence adjacent to the splice site in order to include a consensus splice site in the resulting alignment. We required that each alignment segment have at least three exact bp matches directly adjoining the consensus donor and acceptor splice site in order to exclude this potential artifact. Approximately 90–92% of rice and Arabidopsis transcript alignments met all of these strict validation criteria. One potential source of invalidation here would be due to the use of transcribed sequences from non-*japonica *cultivars of rice or non-Columbia ecotypes for Arabidopsis.

Using PASA, we assembled the 501,379 spliced rice transcript alignments into 43,869 alignment assemblies, 23,308 of which contain FL-cDNAs (Table 1). We also assembled 369,092 spliced Arabidopsis transcript alignments into 29,183 alignment assemblies, of which 22,951 include FL-cDNAs (Table 1). For ESTs that met all other mapping criteria but lack evidence for being spliced (i.e. they are a single exon), we excluded these transcripts from being assembled. This invalidation step will minimize the spurious inclusion of incompletely processed transcripts in the retained intron class. The alignment assemblies, each of which corresponds to at least one distinct mRNA isoform, served as the substrate for this current and comprehensive analysis of alternative splicing in higher plants.

### Distribution of PASA assemblies within isoform clusters

The alignment assemblies were clustered based on overlapping genome coordinates and transcribed orientations in order to group together those assemblies that correspond to the same genomic locus or gene. Each cluster of PASA alignment assemblies can contain either single or multiple transcript alignment assemblies. A cluster comprising multiple assemblies indicates splicing variations mapped to the same genomic locus. A total of 18,506 rice and 16,763 Arabidopsis clusters comprise only a single assembly (i.e. a single transcript isoform), thereby lacking evidence of alternative splicing. However, 8,991 (32.7%) rice clusters have more than one assembly and range from 5,151 clusters having two assemblies to two clusters having 17 distinct assemblies [see Additional file 1]. The results for Arabidopsis are comparable to rice. The majority of clusters supporting alternative splicing contain just two assemblies; 57.3% of the multiple assembly containing clusters for rice and 72.3% in Arabidopsis [see Additional file 1]. The data from both species demonstrate that as the number of assemblies per cluster increases, the corresponding number of clusters decreases precipitously. While alternative splicing can produce a wide variety of isoforms, such as the 38,106 possible forms for the *Drosophila Dscam *gene [30], these data in rice and Arabidopsis clearly indicate that alternative splicing produces only a limited number of isoforms in plants. Given that PASA alignment assemblies in clusters of multiples are indicative of alternative splicing variations, and each assembly is representative of a given (partial or complete) splicing isoform of a gene, we henceforth use the term assembly and isoform interchangeably.

### Description of the alternative splicing isoforms observed in rice and Arabidopsis

**Figure 1:**
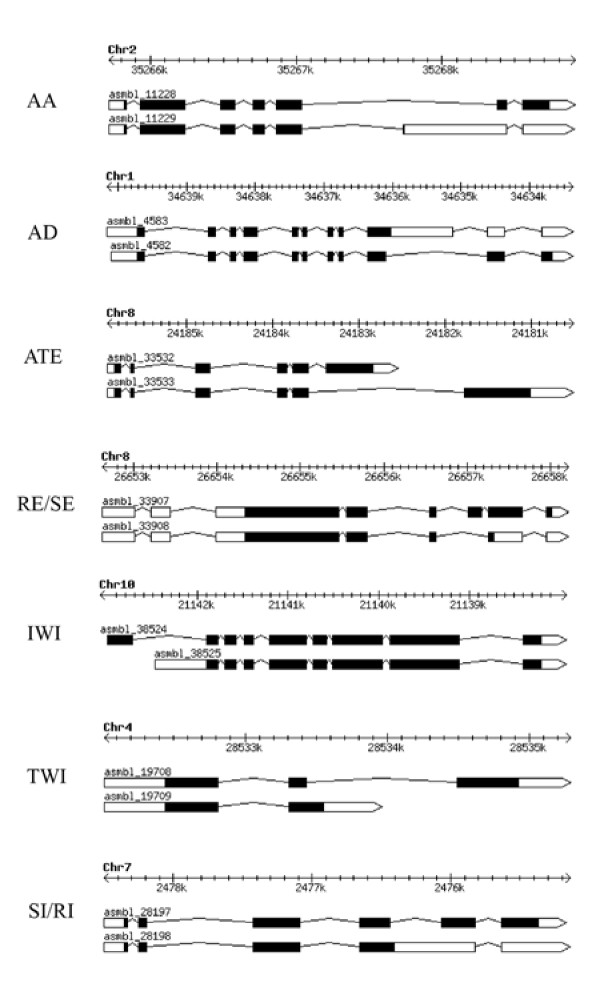
**Diagrams of the nine classes of alternative splicing**. These are: Alternate Acceptor (AA), Alternate Donor (AD), Alternate Terminal Exon (ATE), Retained Exon and Skipped Exon (RE/SE), Initiation within an Intron (IWI), Termination within an Intron (TWI), and Spliced Intron and Retained Intron (RI/SI). The black boxed regions represent the coding region while the white boxed regions represent the 5' and 3' UTR regions. Introns are represented by a single line.

Prior research has shown that the classes of splicing variations observed through comprehensive analyses in Arabidopsis are similar to that observed in mammalian systems [5,12,15,16,31]. We have assigned labels to rice and Arabidopsis splicing isoforms corresponding to the following nine classes of alternative splicing: a) Alternate Acceptor (AA), b) Alternate Donor (AD), c) Alternate Terminal Exon (ATE), d) Skipped Exon (SE), e) Retained Exon (RE), f) Initiation Within an Intron (IWI), g) Termination Within an Intron (TWI), h) Retained Intron (RI), and i) Spliced Intron (SI). Note that the RE and SE classes are reciprocal, i.e. an assembly of the SE class is complemented by its cognate paired assembly displaying the RE class. Likewise, the SI and RI classes are reciprocal. Examples of each of the classes of alternative splicing are illustrated in Figure 1 and described below.

The AA and AD classes involve isoforms with a shared intron, overlap among adjacent exons, but different splice sites chosen for either the donor or the acceptor sites.

The ATE class is defined as having two completely distinct sets of terminal (either 5' or 3') exons that possess no overlapping exon sequence. The RE class includes a distinct exon in one isoform that is spliced out of its corresponding isoform (e.g. the SE isoform). The IWI and TWI classes are conceptually similar where either the 5' or the 3' end of the transcript isoform occurs in an intron of its longer isoform. The final alternative splicing class, RI, is defined by the alternatively spliced isoform retaining an intron that is found spliced in the corresponding SI isoform.

### Relative support for splicing variation(s)

The support for alternative splicing derived from PASA-mediated assemblies can correspond to either a single transcript or multiple independent transcripts. The number of underlying individual transcript alignments exhibiting a specific splicing variation may reflect the *in vivo *prevalence of the variation as compared with the number of transcripts exhibiting a mutually exclusive variation. Caution is needed when only a single transcript supports a variation, as this may reflect a cDNA that was captured prior to the completed processing of the pre-mRNA by the spliceosome, or a possible aberration in transcription [32]. Ratios of the total number of underlying transcripts responsible for each mutually exclusive pair of splicing variations found via pairwise comparisons of isoforms were calculated, and this value allowed us to infer the major and minor isoforms. This ratio is termed the transcript ratio (see Materials and Methods for details). The analysis presented here requires that each variation be supported by two or more transcripts.

**Figure 2:**
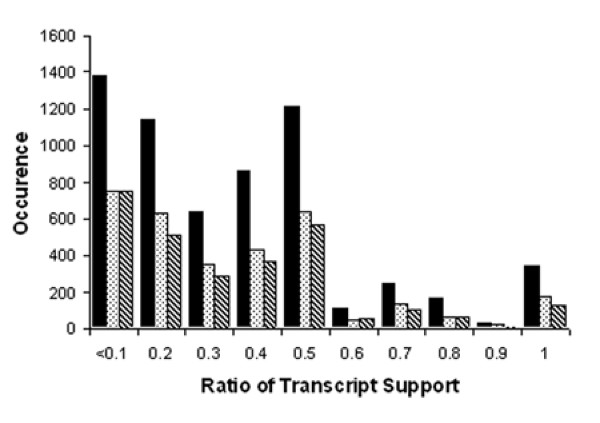
**Histograms displaying the transcript support for three of the alternatively spliced isoforms in rice**. The legend for the histogram is alternate acceptor (black bar), alternate donor (stippled bar), and alternate terminal exon (diagonally hatched).

**Figure 3:**
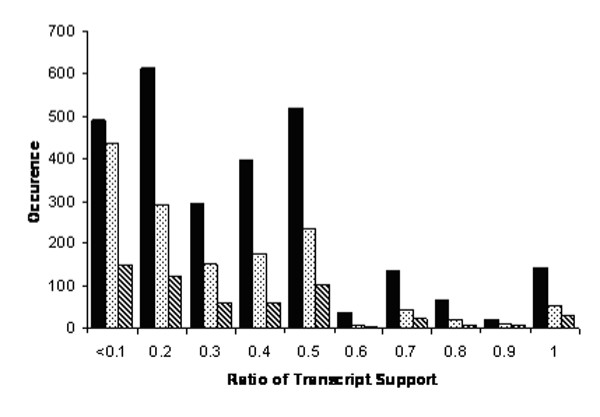
**Histograms displaying the transcript support for three of the alternatively spliced isoforms in Arabidopsis**. The legend for the histogram is alternate acceptor (black bar), alternate donor (stippled bar), and alternate terminal exon (diagonally hatched).

Figure 2 depicts histograms of the ratio of transcript support for the mutually exclusive symmetrical variations found in the AA, AD and ATE classes between isoforms at each locus for rice and Figure 3 contains the parallel data for Arabidopsis. These histograms exclude those variations supported by only one transcript alignment. These histograms have 10 increments ranging from 0.1 to 1 on the x-axis; these increments reflect bins for the transcript ratio with the predominant isoform in the denominator. The y-axis indicates the number of pairwise assembly comparisons that identify these splicing variations as binned by their respective ratios of underlying transcript support. The ratio of the number of transcripts supporting the alternatively spliced isoform to the number of transcripts supporting the predominant isoform is typically less than 0.5. These histograms of the transcript ratios indicate there is clearly a predominant isoform in both rice and Arabidopsis for the majority of the AA, AD and ATE classes.

Unlike the symmetrical classifications of AA, AD and ATE where each isoform variant is given the same label, the remaining variations (RI [RI vs SI], SE [RE vs SE], IWI and TWI) are labeled asymmetrically which allows us to examine the support for each mutually exclusive type and to deduce which are the major and minor variant(s). Table 2 has three bins reflecting the transcript support for each of the remaining six classes of alternative splicing. The three bins of ratios are minor (a ratio less than one), equal/equivalent (a ratio of 1), or major (a ratio greater than one). In both rice and Arabidopsis, the SE, TWI, IWI and RI classes are generally the minor variants when compared to their counterparts. For example, 61.0% of the rice skipped exons are the minor isoforms when compared to their cognate retained exon (RE) isoform. While the IWI and TWI splicing variations may be legitimate isoforms transcribed from alternate promoters or exhibiting alternate 3' termini, they may also result from aberrant transcription or processing or possibly obtained as cDNA cloning artifacts. Data for comparisons of assemblies with all (e.g. including support from only a single transcripts) available transcripts are provided [see Additional file 2].

### Alternative splicing class distribution

For rice and Arabidopsis, the prevalence of the nine alternative splicing classes is shown in Table 3. An event is defined here as the unique feature (e.g. donor site, acceptor site, a series of skipped exon(s), etc) isolated from the assembly comparisons. Overall, 36,650 alterative splicing events were found in rice which translates into alternative splicing in 23,592 (53.8%) rice alignment assemblies, 8,945 (32.5%) of the clusters, and 8,772 (15.7%) of the total TIGR annotated rice genes [4]. A total of 16,252 (40%) alternative splicing events were identified in Arabidopsis within 11,665 (40%) of the Arabidopsis alignment assemblies, 5,155 (23.5%) of the clusters, and 5,313 (17.7%) of the total TIGR annotated Arabidopsis genes [33]. These data show a significant increase of alternative splicing in rice and Arabidopsis from that reported by Wang and Brendel, whose dataset had a total of 369,218 spliced and unspliced Arabidopsis ESTs/cDNAs and 283,816 spliced and unspliced rice ESTs/cDNAs [18].

One caveat required when looking at the breakdown of alternative splicing by class in Table 3 is that the sums of the nine splicing classes exceed the totals presented above. This disparity is due to the fact that a single cluster can contain multiple assemblies [see Additional file 1] and each assembly can contain several of the alternative splicing classes when compared to the other assemblies in the shared cluster. The four most frequently observed alternative splicing classes in both rice and Arabidopsis are AA, SI, RI, and AD, with the AA class being the most frequent. Of the remaining five classes (ATE, TWI, SE, RE and IWI), the percentages for the assembly comparisons are higher in each case for rice than in Arabidopsis but with a similar relative distribution.

When considering the abundance of splicing variations found on a cluster basis, the reciprocal RI/SI and symmetrical AA classes occur most frequently in rice and Arabidopsis. As observed in the alignment assembly statistics, the cluster percentages for each of the remaining classes are higher in rice than in Arabidopsis. Three analyses using EST clustering in Arabidopsis have shown that the RI class was most frequent among the classes of alternative splicing [16,18,34]. Notably, both the prevalence of the TWI and IWI classes are far greater in rice when compared to Arabidopsis. Earlier we noted that the TWI and IWI classes are largely unsupported in comparison to their counterparts that lack these variations. Their accumulation in the rice data may be due to the vast quantity of rice ESTs as compared with FL-cDNAs. Arabidopsis has nearly double the number of FL-cDNAs in the assemblies and the subsequent clusters when compared to rice and these FL-cDNA assemblies presumably provide a more complete representation of the gene, which may be reflected in the lower rates of TWI and IWI in Arabidopsis.

Given that a cluster of assemblies can contain one or more of the alternative splicing isoforms, an interesting parallel in the distribution of alternative splicing in the clusters for the two species can be observed. In both rice and Arabidopsis, the number of discrete alternative splicing classes in a single cluster can range from one to nine [see Additional file 3]. These data show that as the complexity of the splicing isoforms increases in a cluster, the number of clusters decreases.

### Frameshifts are a frequent consequence of chosen alternate acceptor and alternate donor sites

Prior work using a limited set of FL-cDNA transcripts has shown the close proximity of alternative splicing sites in Arabidopsis [35] and this has also been noted in human and mouse [20,22,23,36,37]. The difference in base pairs (bp) between either the alternatively spliced isoforms' donor or the acceptor site was calculated as "delta bp". These delta bp values were analyzed to determine whether the AA or AD splicing isoform would retain its translation frame by being a multiple of three (an integral number of codons) or result in a translational frameshift for those delta bp values that are not a multiple of three. For the 7,071 assembly comparisons that were performed to identify the delta bps for the rice AA class, 2,797 (39.6%) are predicted to retain their translational frame with the remaining 4,274 AA having a translational frameshift downstream of the splice site. For the 3,174 assembly comparisons in the Arabidopsis AA class, 1,491 (47.0%) retain their translational frame and the remaining 1,683 will be frameshifted.

Similar to the AA class, the majority of AD assemblies in both Arabidopsis and rice are predicted to lead to a frameshift when translated. For the 3,795 assembly comparison in rice, 2,717 (71.6%) will lead to a frameshift, and for Arabidopsis, 1,002 of the total 1,635 (61.3%) assembly comparison will lead to a frameshift in translation.

### Distribution of sequence lengths between alternate donors and acceptors

**Figure 4:**
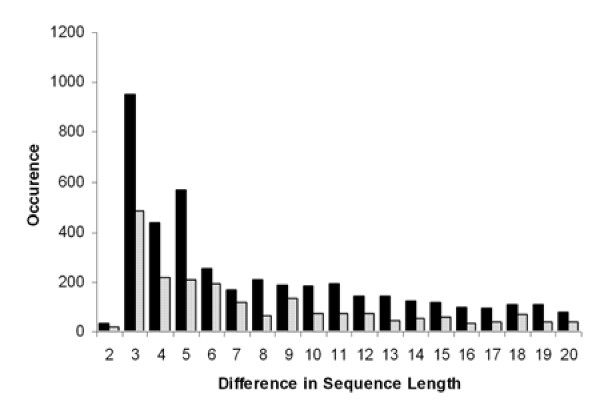
**Histograms displaying the occurrence of the alternate acceptor class by difference in sequence length between the two alternatively spliced acceptor sites**. The histogram shows the bins for the range from 2 bp to 20 bp for rice (black bar) and Arabidopsis (stippled bar).

Histograms were generated after binning all of the common delta bp values for the range between 2 bp and 20 bp. The AA class had a maximal delta bp peak of 3 bp for both rice and Arabidopsis (Figure 4) with a maximal occurrence of 953 alternate site pairs for rice and 485 for Arabidopsis. This maximal peak of 3 bp in the AA class was recently noted in comparative studies of mouse and human alternatively spliced genes [20-22]. For the AA class, the full data set of delta bp for rice and Arabidopsis is provided [see Additional files 4 and 5].

**Figure 5:**
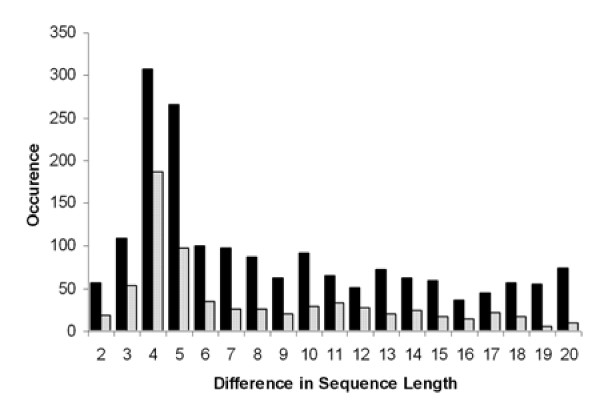
**Histograms displaying the occurrence of the alternate donor class by differences in sequence length between the two alternatively spliced donor sites**. The histogram shows the bins for the range from 2 bp to 20 bp for rice (black bar) and Arabidopsis (stippled bar).

For rice, the AD class has a maximal occurrence over the 4 bp and 5 bp bins with 308 and 265 examples, respectively for rice, and 186 and 97 examples for Arabidopsis, respectively (Figure 5). The entire data set for the AD class is provided [see Additional files 6 and 7]. This maximal peak in AD was noted in the recent work with human and mouse [20,22,23].

### Nucleotide distribution flanking the 3' splice acceptor in rice

**Figure 6:**
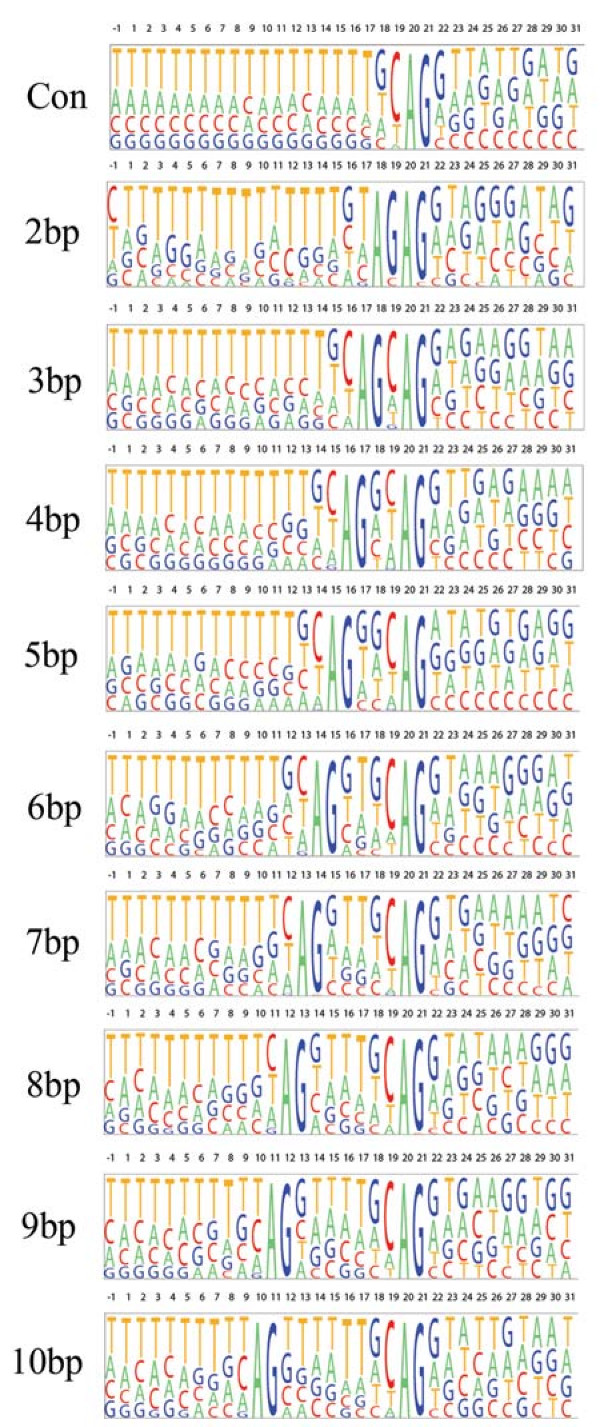
**Pictogram illustrating the nucleotide sequence flanking the 3' splice site acceptor in rice**. This analysis was done using the sequence from the alternate acceptor class with a sequence length difference between the two isoforms ranging from 2 bp to 10 bp. The consensus acceptor site is shown at the top (Con).

Prior work on the acceptor site in the human genome generated the consensus acceptor sequence of (C/T)_n_N(C/T)AG|G [38]. For the AA class with a delta bp value of three, the DNA structure of NAGNAG is observed where either of the AG dinucleotides can be utilized as an acceptor [19]. For mammalian species, the most common DNA sequence for this hexamer is CAGCAG [19,20]. To investigate whether this NAGNAG motif in mammalian species is also present in higher plants, we isolated 32 bp flanking the splice site for each of the delta bp values ranging from two to 10. Pictograms representing the base frequency at each position about the AA variation are displayed in Figure 6 and confirm that the most frequent sequence is a tandem repeat of CAGCAG [39]. The rice consensus acceptor site (T)_n_NCAG|N was generated using 10,000 randomly chosen non-alternatively spliced acceptor splice sites and is shown at the top of Figure 6. Interestingly, as the sequence length increases between the two AG acceptors from 3 bp to 10 bp, a clear pattern emerges where the intervening sequence between the acceptors has a consensus of CAGG(T)_n_GCAG. This feature was observed in the *Drosophila *Sex-lethal gene where the pyrimidine tract was noted for the sequence between alternative acceptors of this gene having a delta bp of 16 bp [40]. Two features germane to the sequences flanking the dinucleotide acceptor are that the intronic sequence upstream of the splice site has a consensus polypyrimidine tract and generally a pyrimidine before the acceptor AG dinucleotide [41]. The exonic (downstream) sequence of the second acceptor splice site has a random distribution of all four nucleotides, reflecting the nucleotide distribution in the mostly protein coding sequence (UTR exons are included in this analysis) [42]. These pictograms show that the distal (downstream) splice site has a consensus CAG site for the acceptor but the distribution of the cytosine nucleotide is reduced in the proximal acceptor as the difference in sequence length between the two sites increases in length.

### Nucleotide distribution flanking the 5' splice donor in rice

**Figure 7:**
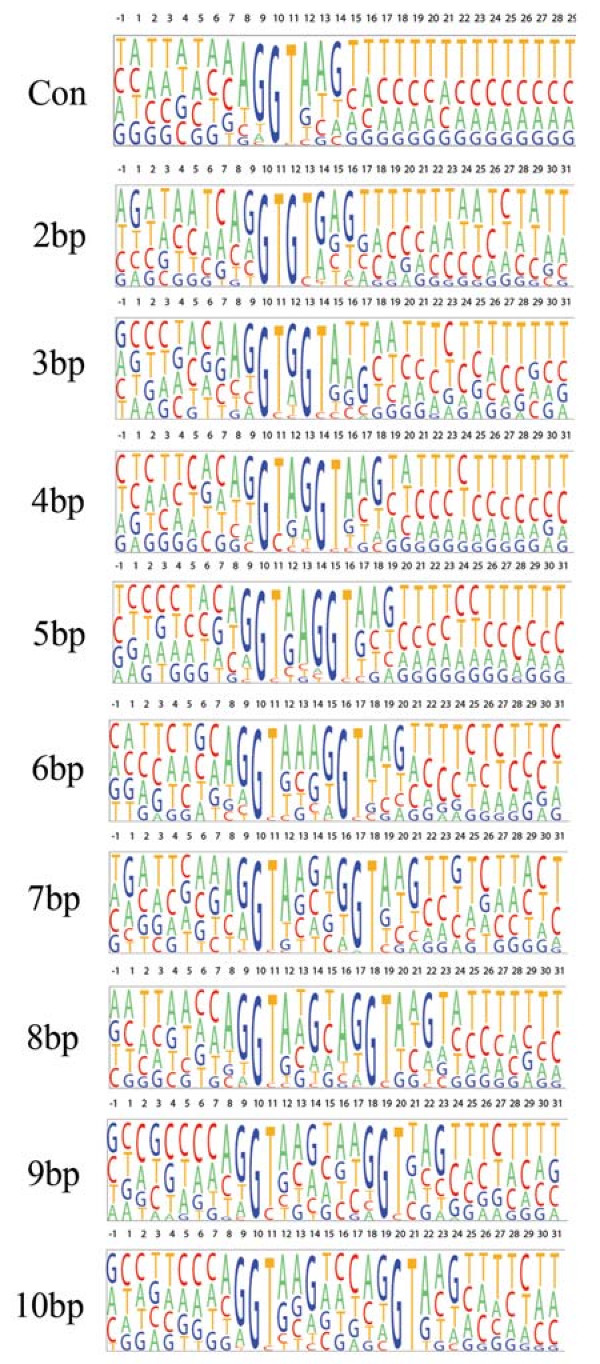
**Pictogram illustrating the nucleotide sequence flanking the 5' splice site donor in rice**. This analysis was done using the sequence from the alternate donor class with a sequence length difference between the two isoforms ranging from 2 bp to 10 bp. The consensus acceptor site is shown at the top (Con).

The DNA sequence that flanks the donor splice site in the AD class with a sequence length difference less than or equal to 10 bp was likewise displayed in pictograms (Figure 7). Previously, a statistical analysis of the sequences flanking donor sites in human revealed a consensus 5' splice site of MAG|GTRAGT with a strong requirement for a guanine nucleotide at positions -1 and +5 [38]. We defined the consensus donor sequence from rice using 10,000 non-alternatively spliced donor sites as AG|GTAWGN. Notably, in all of the pictograms there is a strong preference for a guanine nucleotide adjacent and upstream of the GT donor dinucleotide in either splicing variant. In the intronic sequence downstream of the distal splice site, a polypyrimidine stretch of sequence is noted. Unlike the AA class where a G(T)_n_G consensus sequence was observed, there appears to be no clear consensus in the intervening sequence between the two donor splice sites. However, two conserved features among these donor sites are noted: first, the proximal and distal nucleotides to the donor splice GT dinucleotide are enriched for a purine nucleotide, and second, the thymine base in the GT donor is substituted for a cytosine at an elevated rate.

### Elevated substitution of a GC donor for a GT donor in the AD class

The enrichment of GC donors in alternatively spliced genes has been observed previously in human and *C. elegans *[43,44]. We isolated all of the donor and acceptor dinucleotides in each splicing event for the AD and AA classes and separately isolated all the canonical donor dinucleotides involved directly in either the AA or AD splicing cases [28,29,42]. A Chi-Square analysis for the distribution of the AG and AC acceptor supports the null hypothesis that the AG (P = 0.99) and AC (P = 0.86) acceptor dinucleotides involved as alternative acceptor splice sites are not significantly different from their overall distribution. For the donors, the AT dinucleotide distribution in alternative splicing is similar to that observed in the whole set (P = 0.85) whereas the GC distribution was significantly elevated (GT (P = 0.04) and GC (P = 3.05 × 10^-47^)).

### Localizing splicing variations to coding sequence or untranslated regions of FL-cDNA-based gene structures

FL-cDNAs have proven highly valuable to the improvement of structural annotation of gene models in Arabidopsis [5,17,45]. Likewise, the RIKEN effort to generate a large set of rice FL-cDNAs has greatly improved its structural annotation [13,14]. We have evaluated the nine classes of splicing events in clusters in which at least one alignment assembly incorporates a FL-cDNA. These FL-cDNA assemblies (FL-assemblies) are used to provide reference gene structures to which splicing variations found in other isoforms can be localized to the protein-coding region (CDS) or to untranslated regions (UTRs) flanking the CDS. A total of 11,716 splicing variations were mapped to 5,599 rice clusters containing at least one FL-cDNA reference assembly. For Arabidopsis, 6,250 splicing variations were mapped to 3,909 FL-cDNA clusters containing at least one FL-cDNA reference assemblies.

Table 4 shows the prevalence of the nine splicing events with respect to the rice and Arabidopsis FL-assembly based reference gene structures. For each reference assembly, the longest possible ORF was identified along with the 5' and 3' UTRs, delineating all of the gene structure components. The location of each alternative splicing event was localized to the reference gene structure for both rice and Arabidopsis and was classified based on containment or overlap with the CDS, 5' UTR, and 3' UTR features for the gene. For seven of the nine alternative splicing classes, the majority of the events were contained solely within the CDS. For the AA and AD classes in rice, 69.8% and 62.4% of these splicing events, respectively, are fully contained within the CDS. Similarly in Arabidopsis, 80.6% of the AA and 73.5% of the AD events occur fully within the CDS. These data suggest that the potential frameshift effects, that were noted previously, can have significant effects on the translated amino acid sequence. Similarly for the RI class, 87.2% in rice and 90.1% in Arabidopsis take place fully within the CDS consequently encoding significantly different translational products. Additionally, the RI case may lead to premature translational termination due to the inclusion of an in-frame premature termination codon (PTC) (See the example in Figure 1). For the TWI, IWI, RE and SE alternative splicing classes, the majority are contained fully within the CDS, which can also lead to potentially significant changes in the translated protein. As expected, the IWI class occurs more frequently in the 5' UTR and CDS. In contrast, the TWI class occurs most frequently in the CDS and 3' UTR. Only the ATE and SI classes have a wide distribution across the six possible locations in both rice and Arabidopsis, reflecting the inherent nature of this class to impact either the 5' or 3' end of the transcript with effects that, in either case, can extend into the CDS.

### Evaluating the impacts of splicing on protein structure

A more restrictive analysis was performed to identify the effects of splicing variations on the coding sequence of alternatively spliced genes based solely on FL-assemblies for both rice and Arabidopsis. Only those assemblies generating a complete ORF of greater than 200 amino acids and having the same translational frame for a subset of the CDS were retained for this analysis. In these cases, the FL-assembly encoding the longest ORF was chosen as the reference to which alternate FL-assemblies were compared.

For rice, 639 reference gene structures based on FL-assemblies were compared to an additional 714 FL-assembly based gene structures for rice (Table 5). For the AA and AD splicing events, 72.1% and 61.1% of these events, respectively, changed the frame of the protein, which supports our earlier inference that the majority of the AA and AD events could alter the frame if translated. For these two classes, the protein encoded by the alternative isoform was shortened to 74.7% (AA) or 76.6% (AD) of the longer isoform's protein length. In contrast, the RI class rarely changed the frame (6.1%) of translation, largely because the RI class led to the incorporation of a termination codon in the sequence of the retained intron. By contrast, when the alternative isoform exhibited the spliced intron, this led to a frameshift in each case, suggesting that this spliced intron within the coding sequence had a significant consequence on the translated product. No effects on the frame of translation were observed when isoforms either initiated or terminated transcription in an intron and the data shown previously clearly shows that the vast majority of TWI and IWI class isoforms are the minor isoform suggesting the effects of the splicing events may be more subtle or could represent artifacts in library construction [18]. The ATE class does not lead to a frameshift event and may reflect a clear way to generate proteomic diversity by translating completely different sequences in these terminal exons. Manual inspection revealed that this is common, but cases also exist where the ATE is actually in the 3'UTR and have no effect on translation. In the RE and SE classes, the data suggest that skipping or retaining exons will commonly lead to a frameshift in the translated sequence although these cases are the least frequently observed.

A total of 738 Arabidopsis FL-cDNA supported alternatively spliced assemblies were compared with 829 reference assemblies (Table 5). The data for Arabidopsis are quite similar to that observed in rice, particularly for the most frequently observed classes of alternative splicing (AA, AD, and RI).

## Discussion

This study presents a large scale analysis of alternative splicing profiles in the rice genome. In addition, a parallel analysis in Arabidopsis was performed for comparative purposes. Publicly available FL-cDNAs and ESTs were used with the PASA program to generate maximal transcript alignment assemblies and to cluster the assemblies according to alternative splicing isoforms. PASA-generated clusters having more than one assembly were screened for each of the nine classes of alternative splicing classes. In addition to enumerating the splicing variations found, we examined the underlying transcript supporting evidence to evaluate variations as major or minor isoforms. Finally, we localized splicing variations to the CDS and UTR regions of gene structures and assessed the effects of alternative splicing on the translated products of the isoforms.

When evaluating the clusters, the RI class was the most frequent for both rice (45.1%) and Arabidopsis (47.9%). Previous results in Arabidopsis and rice using an EST clustering strategy showed that the RI class is the most frequent [16,18,34]. However, by excluding the single exon transcripts, we observed fewer RI events (for example 6,887 vs. 7,774 in rice and 3,655 vs. 4,635 in Arabidopsis) than a recent report [18]. By localizing variations to the CDS or UTR of FL-assembly based gene structures, we found the RI isoform to be largely contained in the coding sequence (Table 5), often leading to the inclusion of an in-frame PTC. Comparison of the SI assembly to its RI counterpart shows that the RI assembly has equal or greater transcript support for 43% of isoform pairs in rice and 30% in Arabidopsis. Thus, the RI class is the prevalent isoform in a sizable minority of cases.

The AA class is the second most frequently encountered class in the clusters from both of these species. Studies with mammalian EST clustering strategies revealed that the maximal occurrence of the distance between two alternate acceptor splice sites is 3 bp [20,22,23], which we also observed in both rice and Arabidopsis. A CAG tandem repeat is the most common sequence at these acceptor splice sites, consistent with the reports in mammalian and plant species [18,20]. Additionally, the pictograms reveal a paucity of G nucleotides in the NAGNAG duplication, another feature in agreement with mammalian research [20]. Additional studies will be needed to determine the rates of proximal and distal usage in the NAGNAG motif where the AA events have the maximal occurrence. *In vitro *research on closely spaced tandem AG acceptors has shown that both acceptors are competitive when the distance between them is less than 6 bp and within 19–23 bp of the branch point sequence [46]. The highest occurrences of binned delta bps for the AA class for rice and Arabidopsis range from three bp to six bp which supports the *in vitro *results. However, a propensity toward alternative splicing has also been noted in genes that have unusually large distances (from 40 bp up to 400 bp) from the identified branch point to the acceptor site, and this interim sequence is hypothesized to contain splicing signal motifs [41]. A systematic analysis of AA events in higher plants will be necessary to identify how these closely spaced alternate acceptors are utilized and to compare the results with those from mammalian systems.

The peak occurrence for the length between alternate donor splice sites was found to be 4 bp and the second most frequent value was 5 bp in both plant species. These maximal occurrence values for the AD class have been previously reported in mouse [22,23]. In addition, for mammalian species, the AD distribution was reported to have a very low, ~3 bp, periodicity from 4 bp up to 15 bp [20]. When considering the consensus sequence at the donor splice junction of AG|GTAWGN derived from human consensus (MAG|GTRAGT) with a strong requirement for a guanine at positions -1 and +5 [38], our data supports the hypothesis that this 4 bp peak occurs when either the proximal or the distal GT is used as a donor (Figure 5) [22,23]. Unlike the AA class, there does not appear to be a consensus sequence between the two donor GT dinucleotides aside from a potential enrichment for purine nucleotides. Akerman and Mandel-Gutfreund also hypothesized that a 3 bp difference in sequence length for the AD class would be "infrequent" [20]. However, the occurrence of a 3 bp AD difference in sequence length is equal to or greater than nearly all other length differences observed indicates flexibility in 5' splice site recognition in higher plants. In the AD class, we observed an elevated rate of the donor dinucleotide sequence GC for GT, a trait previously observed in humans and *C. elegans *[43,44]. A survey of AD splicing events across eukaryotes will be needed to determine whether the elevated rates of the GC donor in the AD class are conserved. As with the AA class, using the FL-cDNA dataset, the majority of the AD class splicing events occur within the CDS and the majority of the AD events lead to a translational frameshift.

The transcript support for the AA and AD classes is of particular interest with respect to potential for translational frameshift. The AA class occurs about twice the rate of the AD class. However, the transcript support histograms have a very similar profile. These histograms show that, while the alternative isoform is less frequent in nearly all cases, these alternative isoforms are neither rare nor isolated. The possibility of a frameshift in the majority of events having small differences in length indicate that the AA and AD classes in both rice and Arabidopsis compare with the results in mouse [43,44]. Using the FL-cDNA as a reference for delineating the likely translated ORF, seven of the nine classes of alternative splicing occur predominantly in the coding sequence and these events have a significant effect on the translated protein. Prior work in other eukaryotic systems has suggested that alternatively spliced transcripts having a PTC are removed by non-sense mediated decay (NMD) [32]. In mammals, degradation of PTC containing mRNAs is induced by the presence of a PTC 50 bp (or more) upstream of the exon/exon boundary in a spliced mRNA [47]. Signals leading to decay occur at the time of translation where exon junction complexes are displaced during translation and the presence of a termination codon before an exon junction induces NMD of the transcript [48,49]. NMD is observed in all eukaryotic species that have been examined and these PTC-containing mRNAs are rapidly degraded [50,51]. A recent report suggested that nearly one-third of all alternative splicing events in human meet the criteria for NMD-mediated degradation and this phenomenon was termed regulated unproductive splicing and translation (RUST) [32]. The conservation of RUST in higher plants has not been described in any detail yet. However the presence of an NMD-like pathway in plants was first suggested by studies showing reduced stability of mRNAs containing PTCs. Studies on mutants of *waxy *mRNA containing PTCs in rice (*Oryza sativa*) have suggested that splicing of the first intron present upstream of PTC is important for NMD of mutant *waxy *mRNA [52]. Also, nonsense but not missense mutants of *xantha *mRNA in barley (*Hordeum vulgare*) appear to be subjected to rapid degradation, even though the mutant mRNA contains PTC in the last exon [53]. Despite these findings, the mechanism of NMD in plants and its role in plant growth and development have not been clarified. In addition, there has been a recent study on the function of plant UPFs which showed that UPF3 of Arabidopsis suppresses aberrantly spliced mRNAs containing PTCs [54].

Interestingly, when restricting the alternative splicing analysis to the UTRs, the AA, AD, RI and SE classes are found to occur far more frequently in the 5'UTR than in the 3' UTR. Given that EST coverage is known to be 3' biased due to the nature of cDNA library generation, the 5'UTR bias for alternative splicing is notable. Following the rule for NMD, these 5'UTR localized events are assumed not to be involved in this post-transcriptional control pathway nor altering (directly) the coding sequence. A recent report using EST alignments in human also identified an enrichment of alternative splicing in the 5'UTR compared to the 3' UTR and the authors suggested this may represent a method for translational control [55,56].

## Conclusion

Alternative splicing has also been proposed as a mechanism to expand the proteomic diversity within a genome. As the EST collections for rice and Arabidopsis have dramatically increased, successive reports have greatly expanded the number of identified alternative splicing isoforms. The data presented here suggest that, while alternative splicing may produce novel translated combinations of the primary amino acid sequence, there is also a large number of transcripts produced by the AA, AD, and RI classes having either frameshifts or PTCs that may have a role in post-transcriptional regulation. These new data will need to be explored via experimentation to determine whether RUST and NMD-mediated pathways are degrading these alternatively spliced transcripts.

## Methods

### Downloading the ESTs and mRNAs

All publicly available ESTs and mRNA sequences for *Oryza sativa *and *Arabidopsis thaliana *(no distinction was made for cultivars or ecotypes) were obtained from the public databases, primarily GenBank, but in the case of rice, supplemented with an EST data set newly released to DDBJ [2,3]. Special care was taken to exclude sequences from RefSeq [57], given that most are not experimentally derived and result in many cases from predicted genes in completed genomes.

### Application of the PASA pipeline to rice and Arabidopsis

The PASA pipeline was used as previously described [5] with the following modifications. Earlier application of PASA utilized the BLAT [58] and sim4 [59] transcript alignment software. Here, we chose instead to use the GMAP software [24], due to its improved accuracy, speed, and impressively low memory requirements. All transcripts, including ESTs and FL-cDNAs were aligned to the genome, followed by our transcript alignment validation procedure and subsequent alignment assembly. Only near perfect alignments were utilized in the alignment assembly process, requiring at least 95% identity across at least 90% of the transcript length. In addition, all internal alignment boundaries were required to adjoin consensus splice sites. In addition to the GT-AG and GC-AG introns, we extended our validation criteria to allow the more rare AT-AC introns. In the case of candidate AT-AC introns, we required that the AT donor site match the extended conserved donor site consensus sequence ATatcc (extended consensus shown in lower case). All other combinations of donors and acceptors were disallowed. The assembly of alignments via the PASA alignment assembly algorithm, and subsequent clustering of overlapping alignment assemblies with same transcribed orientation were performed exactly as previously described. All clusters of PASA assemblies correspond to single-linkage clusters of alignment assemblies having identical transcribed orientations and overlapping by at least 50% of either alignment's genome span. Clusters of alignment assemblies were mapped to existing genome annotations as previously described, using a 1% overlap criteria instead of the original 40% employed.

### Labeling of alternative splicing variations

Each cluster of PASA alignment assemblies containing more than one alignment assembly was a direct result of at least one splicing variation. An all-vs-all comparison was performed among each pair of alignment assemblies within each cluster. An individual alignment assembly was labeled as having a specific splicing variation as evident when compared to another alignment assembly supporting the variation. For example, an alignment assembly was labeled as having a retained intron only after it was compared to another alignment assembly that had this specific intron spliced. Likewise, an alignment assembly was labeled as having an alternative acceptor site when compared to another alignment assembly that exhibited the corresponding exon with a different acceptor site at its boundary. Most variations were labeled reciprocally, including AD, AA, ATE, RI, and SE. In the case of an AA or AD splice site, and in the case of ATE isoforms, both alignment assemblies exhibit the corresponding variation, and each is labeled with this exact property. In the case of RI or SE, one assembly has this property, but the other assembly that provides evidence for the retained intron or skipped exon in former, has the converse property, and is labeled accordingly. The label serving as the counterpart to SE is RE, and the counterpart to RI is SI. However, the variations that initiate or terminate within intron (IWI or TWI) are asymmetrically labeled; only the specific assembly that exhibited the property was given the corresponding label. Labeling isoforms in this way is useful for the purpose of easily identifying isoforms with a given class of splicing variation, or to identify isoforms that exhibit a combination of splicing variations.

The individual transcripts within an alignment assembly that provide supporting evidence for individual splicing variation (label) were identified as those transcripts within an assembly that were disjoint from the assembly being compared and found to overlap the coordinates of the splicing variation. This comparison was performed reciprocally, regardless of whether the label was symmetrical or asymmetrical, so that for every splicing variation identified, the supporting evidence for each mutually exclusive variation was captured. We calculated the ratio of transcript support for each variation and used this ratio to infer the major and minor splice variants. The combination of labels assigned to an isoform, the genome coordinates of the localized variation, and the identity of the transcripts supporting each variation were stored in a MySQL database.

### Algorithms for identifying and classifying splicing variations

Paired isoforms (A, B), found in the same cluster of PASA assemblies, and found to overlap each other, were examined as follows:

• AA and AD: All exons of isoform A are mapped to exons of isoform B based on genome coordinates. Alternate donor or acceptor splice sites are identified where one-to-one mappings exist between exons having non-identical coordinates at internal splice junctions.

• RI: Introns of isoform B are compared to the exons of isoform A. An intron of isoform B completely encapsulated by an exon of isoform A is identified and classified as a retained intron in isoform A. Isoform B is reciprocally labeled as having the spliced intron variation.

• SE: Exons of isoform A are compared to introns of isoform B. Exons of isoform A found completely encapsulated by a single intron of isoform B, but having neighboring exons on both sides that anchor to exons in isoform B, are classified as retained exons. A single 'retained exons' classification can contain one or more neighboring exons, all localized to a single intron of isoform B. Isoform B is reciprocally labeled as having 'skipped exons' that localize to one of its introns.

• ATE: Exons of isoform A are mapped to exons of isoform B based on genome coordinates. In a search for overlapping exons between isoform A and isoform B starting from either termini, if the first overlapping exon found between isoform A and B is not a terminal exon in either isoform, the series of exons from the corresponding termini of isoform A found prior to the first overlapping exon are grouped and classified as alternate terminal exons (a single alternate terminal event).

• IWI and TWI: The initial or terminal exon of isoform A begins or ends in an intron of isoform B and overlaps an exon of isoform B. Although we use the terms initiation within intron (IWI) and termination within intron (TWI), this simply indicates that our transcript alignment evidence begins or ends within an intron and is not necessarily indicative of transcriptional processes involving either transcriptional initiation, termination, or polyadenylation sites that may be responsible for these variations.

### Identification of alternative acceptor and alternative donor delta bp values

All pairs of isoforms exhibiting the AA splice site or AD splice site (isoforms specifically labeled with these variations as described above) were examined. For each pair of symmetrically labeled isoforms, the length between the alternate splice sites was computed, assigned as the delta value, binned and counted accordingly.

### Localizing splicing variations to CDS regions or UTRs

Localizing a splicing variation to the CDS or UTR requires that we are highly confident in the location of these features. Confidence in this matter is based on the accuracy of the underlying gene structure. We cannot rely solely on existing rice or Arabidopsis gene structure annotations because in many cases, the genes are largely predicted by *ab initio *gene prediction programs, and we cannot confidently ascertain the accuracy of these gene predictions. We can, however, have a high degree of confidence in gene structures that are supported by FL-cDNAs; those cDNAs that encode both the complete protein-coding structure of the gene and extended UTR regions. Consequently, we limit our localizations of splicing variations to clusters of PASA assemblies including at least one FL-cDNA.

In such a cluster of PASA alignment assemblies, we chose a FL-cDNA that was found to encode a complete gene structure such that a full-length ORF was found with both a start and a stop codon. In cases where multiple candidate full-length assemblies existed, we chose the entry with the longest complete ORF. All other assemblies in this cluster were compared to this reference gene structure, and the positions of splicing variations were mapped to the CDS, the 5' UTR, or the 3' UTR.

### Analysis of the effects of splicing variation on protein structure

Similarly to the above localization of splicing variations, where we required a high level of confidence in the underlying gene structure, here we require a high level of confidence in the complete gene structures for multiple isoforms of a single gene, so that we can perform meaningful comparisons between isoforms and ascertain the effects of the splicing variation(s) on the primary structure of the protein. Our analysis was hereby confined to those clusters of PASA alignment assemblies including multiple assemblies considered to be full-length (assemblies including at least one FL-cDNA). The protein isoform lengths were compared, and their gene structures were examined for the presence of a change in reading frame.

### The enhanced PASA software

Enhancements to the PASA software resulting from our efforts described here include:

• the capability to utilize GMAP for mapping and aligning transcripts to the genome, and as an alternative to blat coupled with sim4.

• the consensus splice site requirement was extended to include the U12-type AT-AC introns.

• the transcript alignment validation procedure can verify perfect sequence matches between the genome and the transcript sequence at *n*-number of bp adjoining the tentative splice junction (default for *n *is three bp, as employed here).

• automated identification and classification of splicing variations found between clustered PASA alignment assemblies.

• CGI-scripts that provide for web-browser based navigation of the results from the mining of alternative splicing variations, including data tables and images that provide illustrations of splicing graphs, localizations of splicing variations, and supporting transcript alignments.

The latest version of the PASA software is available [60] and distributed under the Open Source Initiative's Artistic License [61].

The data sets and analysis generated in this study are available upon request.

## Authors' contributions

MAC coordinated the analysis, generated figures and tables, and drafted the manuscript. BJH enhanced the PASA software, ran the PASA analysis, generated the summary data, produced the pictograms, and helped to draft the manuscript. JPH assisted with data analysis and development of the figures. SMM participated in manuscript development. CRB participated in the coordination of the analysis and helped draft the manuscript. All authors have read and approved the final manuscript.

## Supplementary Material

Additional file 1 Distribution of assemblies per cluster. Click here for file

Additional file 2 Transcript support data for all alternative splicing classes including those with one transcript. Click here for file

Additional file 3 Distribution of alternative splicing isoforms per cluster. Click here for file

Additional file 4 Coordinates for the alternative acceptor splicing sites in the two isoforms for rice. Click here for file

Additional file 5 Coordinates for the alternative acceptor splicing sites in the two isoforms for Arabidopsis. Click here for file

Additional file 6 Coordinates for the alternative donor splicing sites in the two isoforms for rice. Click here for file

Additional file 7 Coordinates for the alternative donor splicing sites in the two isoforms for Arabidopsis. Click here for file

## Figures and Tables

**Table 1:** Summary statistics are given for the PASA-generated assemblies in rice and Arabidopsis.

**Number**	**Rice**	**Arabidopsis**
Total ESTs	1,156,705	690,119
Total mRNAs	35,078 (33,799 FL)	66,642 (61,117 FL)
Total Transcripts	1,203,577	690,119
Survive Cleaning	1,190,502 (33,797 FL)	683,137 (61,113 FL)
Map to Genome	1,149,730 (32,349 FL)	671,774 (61,085 FL)
Valid Alignments	1,055,860 (30,099 FL)	603,234 (56,668 FL)
Excluding Single Exon Transcripts	501,379 (30,099 FL)	369,092 (56,668 FL)
PASA alignment assemblies	43,869 (23,308 FL)	29,183 (22,951 FL)
PASA assembly clusters	27,497 (19,888 FL)	21,922 (18,701 FL)

**Table 2:** Ratios of the number of transcripts supporting the alternative splicing classes of skipped exon (SE), initiation within intron (IWI), termination within intron (TWI), and retained intron (RI).

**Variation**	**Prevalence**	**Rice**	**Arabidopsis**
SE	Minor	1,603 (61.0%)	284 (54.2%)
	Equal	118 (4.5%)	28 (5.3%)
	Major	909 (34.5%)	212 (40.5%)
IWI	Minor	3,570 (94.5%)	763 (90.6%)
	Equal	61 (1.6%)	21 (2.5%)
	Major	146 (3.9%)	58 (6.9%)
TWI	Minor	3,988 (94.8%)	804 (93.9%)
	Equal	58 (1.4%)	14 (1.6%)
	Major	162 (3.8%)	38 (4.5%)
RI	Minor	7,713 (70.0%)	3,574 (76.6%)
	Equal	546 (5.0%)	163 (3.5%)
	Major	2,755 (25.0%)	926 (19.9%)

These counts and ratios were calculated from pairwise comparisons between PASA assemblies. Cases where only a single transcript supports a variation were excluded. For each variation type, SE, IWI, TWI and RI, the prevalence of evidence indicates that it is the minor variation for the majority of cases examined. Note that the cognate isoform for SE is the retained exon (RE) and the cognate isoform for RI is the spliced intron (SI).

**Table 3:** Summation of the distribution of the nine alternative splicing classes by events, assembly, cluster and gene for rice and Arabidopsis.

**Rice**				
**Isoform**	**Events**	**Assemblies**	**Clusters**	**Genes**

AA	6,823 (18.6%)	8,171 (34.6%)	2,840 (31.8%)	2,838 (32.4%)
AD	3,604 (9.8%)	4,652 (19.7%)	1,599 (17.9%)	1,619 (18.5%)
ATE	4,188 (11.4%)	4,227 (17.9%)	1,417 (15.8%)	1,503 (17.1%)
SE	1,339 (3.7%)	1,707 (7.2%)	1,142 (12.8%)	1,119 (12.8%)
RE	1,496 (4.1%)	1,957 (8.3%)	1,142 (12.8%)	1,128 (12.9%)
IWI	2,444 (6.7%)	2,513 (10.7%)	2,139 (23.9%)	2,085 (23.8%)
TWI	2,982 (8.1%)	3,141 (13.3%)	2,543 (28.4%)	2,475 (28.2%)
SI	6,887 (18.8%)	7,592 (32.2%)	4,035 (45.1%)	3,933 (44.8%)
RI	6,887 (18.8%)	5,579 (23.7%)	4,035 (45.1%)	3,924 (44.7%)

Totals	36,650	23,592	8,945	8,772

**Arabidopsis**				

**Isoform**	**Events**	**Assemblies**	**Clusters**	**Genes**

AA	3,885 (23.9%)	4,220 (36.2%)	1,720 (33.4%)	1,793 (33.8%)
AD	1,854 (11.4%)	2,179 (18.7%)	865 (16.8%)	906 (17.1%)
ATE	900 (5.5%)	913 (7.8%)	340 (6.6%)	390 (7.3%)
SE	396 (2.4%)	442 (3.8%)	348 (6.8%)	357 (6.7%)
RE	415 (2.6%)	486 (4.2%)	348 (6.8%)	361 (6.8%)
IWI	722 (4.4%)	729 (6.3%)	685 (13.3%)	695 (13.1%)
TWI	770 (4.7%)	794 (6.8%)	713 (13.8%)	743 (14.0%)
SI	3,655 (22.5%)	3,667 (31.4%)	2,467 (47.9%)	2,521 (47.5%)
RI	3,655 (22.5%)	2,945 (25.3%)	2,467 (47.9%)	2,510 (47.2%)

Totals	16,252	11,665	5,155	5,313

Percentages are based upon the total counts shown below. They do not include those assemblies, clusters, and genes not implicated in alternative splicing. An event is defined as the occurrence of one of the nine isoforms within an assembly. An assembly derived from overlapping EST and FL-cDNA alignments can contain more than one event.

**Table 4:** Using the annotation derived from FL-cDNAs, the alternative splicing effects are classified relative to the CDS and UTRs.

**Rice**									
**Type**	**AA**	**AD**	**ATE**	**SE**	**RE**	**IWI**	**TWI**	**SI**	**RI**

CDS contained	1,508	679	390	311	184	1,224	1,353	351	2,111
CDS overlaps UTRs	1	1	113	75	2	0	0	342	0
3' UTRs contained	183	71	47	21	25	34	147	416	146
3' UTR overlaps CDS	1	1	49	23	0	0	0	246	0
5' UTR contained	468	336	200	80	65	136	84	367	165
5' UTR overlaps CDS	0	0	178	56	2	0	0	98	0

Totals	2,161	1,088	977	566	278	1,394	1,584	1,820	2,422

**Arabidopsis**									

**Type**	**AA**	**AD**	**ATE**	**SE**	**RE**	**IWI**	**TWI**	**SI**	**RI**

CDS contained	1,206	509	97	138	65	330	441	211	1,789
CDS overlaps UTRs	0	0	37	26	0	0	0	106	0
3' UTRs contained	56	24	4	3	1	6	49	74	57
3' UTR overlaps CDS	0	0	11	4	0	0	0	63	0
5' UTR contained	234	160	58	24	20	86	16	202	140
5' UTR overlaps CDS	0	0	109	22	0	0	0	43	0

Totals	1,496	693	316	217	86	422	506	699	1,986

The categories are mutually exclusive in this analysis (i.e. those isoforms in one category are not present in any of the other five categories).

**Table 5:** Frame change and protein truncation statistics by class for FL-cDNAs supported assemblies within the same cluster.

**Rice**			
**Isoform**	**Total**	**No. with Frame Changes**	**Average % of protein length**

AA	61	44 (72.1%)	74.7
AD	36	22 (61.1%)	76.6
ATE	28	0 (0%)	83.9
SE	9	9 (100%)	56.4
RE	11	5 (45.5%)	79.2
IWI	33	0 (0.0%)	77.2
TWI	127	0 (0.0%)	69.1
SI	14	14 (100%)	77.6
RI	165	10 (6.1%)	73.1

**Arabidopsis**			

**Isoform**	**Total**	**No. with Frame Changes**	**Average % of protein length**

AA	120	94 (78.3%)	73.6
AD	62	45 (72.6%)	73.4
ATE	7	0 (0%)	70.1
SE	5	5 (100%)	75.1
RE	5	0 (0%)	80.0
IWI	20	0 (0%)	76.8
TWI	66	0 (0%)	72.0
SI	23	23 (100%)	74.0
RI	302	20 (6.6%)	77.2

This analysis is restricted to those isoforms displaying only a single class of alternative splicing when compared to the gene structure.
